# Broad and Differential Animal Angiotensin-Converting Enzyme 2 Receptor Usage by SARS-CoV-2

**DOI:** 10.1128/JVI.00940-20

**Published:** 2020-08-31

**Authors:** Xuesen Zhao, Danying Chen, Robert Szabla, Mei Zheng, Guoli Li, Pengcheng Du, Shuangli Zheng, Xinglin Li, Chuan Song, Rui Li, Ju-Tao Guo, Murray Junop, Hui Zeng, Hanxin Lin

**Affiliations:** a Institute of Infectious Disease, Beijing Ditan Hospital, Capital Medical University, Beijing, China; b Beijing Key Laboratory of Emerging Infectious Disease, Beijing, China; c Department of Biochemistry, Western University, London, Ontario, Canada; d Baruch S. Blumberg Institute, Hepatitis B Foundation, Doylestown, Pennsylvania, USA; e Department of Pathology and Laboratory Medicine, Western University, London, Ontario, Canada; Loyola University Chicago

**Keywords:** SARS-CoV-2, animal ACE2, receptor, entry, furin cleavage, animal hosts

## Abstract

The COVID-19 pandemic has caused an unprecedented global public health and economic crisis. The origin and emergence of its causal agent, SARS-CoV-2, in the human population remains mysterious, although bat and pangolin were proposed to be the natural reservoirs. Strikingly, unlike the SARS-CoV-2-like coronaviruses (CoVs) identified in bats and pangolins, SARS-CoV-2 harbors a polybasic furin cleavage site in its spike (S) glycoprotein. SARS-CoV-2 uses human angiotensin-converting enzyme 2 (ACE2) as its receptor to infect cells. Receptor recognition by the S protein is the major determinant of host range, tissue tropism, and pathogenesis of coronaviruses. In an effort to search for the potential intermediate or amplifying animal hosts of SARS-CoV-2, we examined receptor activity of ACE2 from 14 mammal species and found that ACE2s from multiple species can support the infectious entry of lentiviral particles pseudotyped with the wild-type or furin cleavage site-deficient S protein of SARS-CoV-2. ACE2 of human/rhesus monkey and rat/mouse exhibited the highest and lowest receptor activities, respectively. Among the remaining species, ACE2s from rabbit and pangolin strongly bound to the S1 subunit of SARS-CoV-2 S protein and efficiently supported the pseudotyped virus infection. These findings have important implications for understanding potential natural reservoirs, zoonotic transmission, human-to-animal transmission, and use of animal models.

**IMPORTANCE** SARS-CoV-2 uses human ACE2 as a primary receptor for host cell entry. Viral entry mediated by the interaction of ACE2 with spike protein largely determines host range and is the major constraint to interspecies transmission. We examined the receptor activity of 14 ACE2 orthologs and found that wild-type and mutant SARS-CoV-2 lacking the furin cleavage site in S protein could utilize ACE2 from a broad range of animal species to enter host cells. These results have important implications in the natural hosts, interspecies transmission, animal models, and molecular basis of receptor binding for SARS-CoV-2.

## Abstract (precis)

SARS-CoV-2 uses human ACE2 as a primary receptor for host cell entry. Viral entry mediated by the interaction of ACE2 with spike protein largely determines host range and is the major constraint to interspecies transmission. We examined the receptor activity of 14 ACE2 orthologs and found that wild-type and mutant SARS-CoV-2 lacking the furin cleavage site in S protein could utilize ACE2 from a broad range of animal species to enter host cells. These results have important implications in the natural hosts, interspecies transmission, animal models, and molecular basis of receptor binding for SARS-CoV-2.

## INTRODUCTION

Coronavirus disease 2019 (COVID-19) was first identified in December 2019 in the city of Wuhan, China (1), and has since spread worldwide, causing ∼2.3 million infections and around 160,000 fatalities as of 18 April 2020 (https://coronavirus.jhu.edu/map.html). These numbers are still growing rapidly. The global COVID-19 pandemic has caused an unprecedented public health and economic crisis.

COVID-19 is caused by a novel coronavirus (CoV), severe acute respiratory syndrome coronavirus 2 (SARS-CoV-2; initially named 2019-nCoV) (2, 3). The origin of SARS-CoV-2 and its emergence in the human population remain mysterious. Many of the early cases were linked to the Huanan seafood and wild-animal market in the city of Wuhan, raising the possibility of zoonotic origin (4). Sequencing analyses showed that the genome of SARS-CoV-2 shares 79.5%, 89.1%, 93.3%, and 96.2% nucleotide sequence identity with that of human SARS-CoV, bat CoV ZC45, bat CoV RmYN02, and bat CoV RaTG13, respectively, suggesting that SARS-CoV-2 probably has bat origins (2, 3, 5). This finding is not surprising as bats are notorious for serving as the natural reservoirs for two other deadly human coronaviruses (hCoVs), SARS-CoV and Middle East respiratory syndrome coronavirus (MERS-CoV), which previously caused global outbreaks (6, 7).

**FIG 1:**
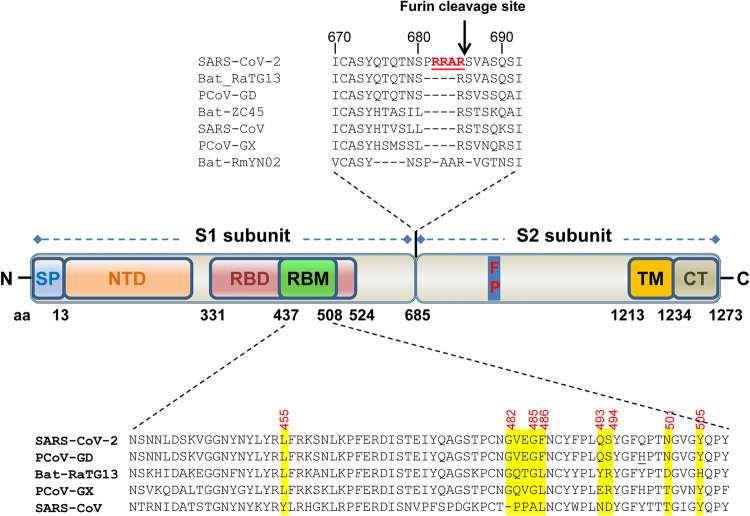
Schematic diagram of domain structures and critical ACE2-binding residues of the spike (S) protein of SARS-CoV-2. The S protein is cleaved into S1 and S2 subunits during biogenesis at the polybasic furin cleavage site (RRAR↓), which is not present in SARS-CoV and other animal SARS-CoV-2-like CoVs. The S1 subunit is required for binding to ACE2 receptor, while the S2 subunit containing a fusion peptide mediates membrane fusion. In SARS-CoV-2, the S1 subunit contains an N-terminal domain and an independently folded domain known as the RBD, which harbors a region called the receptor binding motif (RBM), that is primarily in contact with receptor. The most critical hACE2-binding residues in the RBM of several SARS-CoV-2-related CoVs are highlighted in yellow and inferred from the crystal structure of RBD/hACE2 complex (16). The only difference in the RBMs between PCoV-GD and SARS-CoV-2 is Q498H (underlined). The GenBank numbers for these CoVs are as follows: SARS-CoV-2 isolate Wuhan-Hu-1, MN908947; SARS-CoV isolate Tor2, NC_004718.3; bat ZC45, MG772933.1; bat RaTG13, MN996532.1; PCoV-GX isolate P4L, MT040333.1; PCoV-GD isolate MP789, MT084071.1. SP, signal peptide; NTD, N-terminal domain; RBD, receptor-binding domain; RBM, receptor-binding motif; FP, fusion peptide; TM, transmembrane domain; CT, cytoplasmic tail; PCoV-GX, pangolin CoV isolate GX-PL4; PCoV-GD, pangolin CoV isolate MP789.

Although SARS-CoV-2 may have originated from bats, bat CoVs are unlikely to jump directly to humans due to a general ecological separation. Other mammal species may have served as intermediate or amplifying hosts whereby the progenitor virus acquires critical mutations for efficient zoonotic transmission to human. This has been seen in the emergence of SARS-CoV and MERS-CoV where palm civet and dromedary camel act as the respective intermediate hosts (7). The Huanan seafood and wild-animal market in the city of Wuhan would otherwise be a unique place to trace any potential animal source; however, soon after the disease outbreak, the market was closed, and all of the wild animals were cleared, making this task very challenging or even impossible. As an alternative, broad screening of wild animals becomes imperative. Several recent studies identified multiple SARS-COV-2-like CoVs (SL-CoVs) from smuggled Malayan pangolins in China. These pangolin CoVs (PCoVs) form two phylogenetic lineages, PCoV-GX and PCoV-GD (8–11). In particular, lineage PCoV-GD was found to carry a receptor-binding motif (RBM) in the spike (S) protein that is nearly identical to that of SARS-CoV-2 (Fig. 1). However, the genomes of these pangolin SL-CoVs share only 85.5% to 92.4% nucleotide identities with the genome of SARS-CoV-2. This is in contrast to SARS-CoV and MERS-CoV for which CoVs isolated from the intermediate hosts palm civet and dromedary camel share 99.6% and 99.9% % genome sequence identities, respectively, with their human counterpart (12, 13). Therefore, pangolins tested in these studies are not the direct intermediate hosts for SARS-CoV-2. Whether or not SARS-CoV-2 came from other pangolins or other wild-animal species remains to be determined.

S protein-driven cellular entry, triggered by receptor recognition, is the major determinant of host range, cell, tissue tropism, and pathogenesis of coronaviruses (14). The S protein of SARS-CoV-2 is a type I membrane glycoprotein, which can be cleaved into S1 and S2 subunits during biogenesis at the polybasic furin cleavage site (RRAR) (Fig. 1) (15–18). Previous studies have shown that furin cleavage is not essential for coronavirus-cell membrane fusion but enhances cell-to-cell fusion (19–23), expands coronavirus cell tropism (24), and increases the fitness of sequence variants within the quasispecies population of bovine CoV (25). Recent studies indicated that cleavage at the S1/S2 boundary by furin in virus-producing cells is a critical primary step that facilitates conformation change triggered by receptor binding during virus entry and subsequent fusion-activating cleavage at the S2′ site, which is located immediately upstream of fusion peptide in the S2 subunit (18, 24, 26). Also, furin cleavage in hemagglutinin (HA) was found to convert an avirulent avian influenza virus isolate into a highly pathogenic isolate (27). Interestingly, this cleavage site is not present in the S protein of SARS-CoV, bat SL-CoVs, or pangolin SL-CoVs identified so far (5, 15). In addition to furin-mediated cleavage in virus-producing cells, SARS-CoV-2 S protein is also cleaved for fusion activation by the cell surface protease TMPRSS2 and lysosomal proteases, e.g., cathepsin L, during virus entry of target cells (15, 18).

During cell entry, S1 binds to the cellular receptor, subsequently triggering a cascade of events leading to S2-mediated membrane fusion between host cells and coronavirus particles (28). S1 protein contains an independently folded domain called the receptor binding domain (RBD), which harbors an RBM that is primarily involved in contact with the receptor (Fig. 1). Human angiotensin-converting enzyme 2 (hACE2) has been identified as the cellular receptor for both SARS-CoV-2 (3, 15, 17, 29) and SARS-CoV (30). In addition to hACE2, ACE2 from horseshoe bat (Rhinolophus alcyone) was found to support cell entry of SARS-CoV-2 S-mediated vesicular stomatitis virus (VSV)-based pseudotyped virus (15). By using infectious virus, it has also been shown that ACE2s from Chinese horseshoe bat (Rhinolophus sinicus), civet, and swine, but not mouse, could serve as functional receptors (3). However, in this infection system, the entry step was coupled with other steps during virus life cycle, i.e., viral genome replication, translation, virion assembly, and budding, and thus the receptor activity of these animal ACE2 orthologs was not directly investigated.

In an effort to search for potential animal hosts, we examined the receptor activity of ACE2s from 14 mammal species, including human, rhesus monkey, Chinese horseshoe bat (Rhinolophus sinicus), Mexican free-tailed bat (Tadarida brasiliensis), rat, mouse, palm civet, raccoon dog, ferret badger, hog badger, canine, feline, rabbit, and pangolin for SARS-CoV-2 and a mutant virus lacking the furin cleavage site in the S protein. Our results show that multiple animal ACE2 proteins could serve as receptors for SARS-CoV-2 and the SARS-CoV-2 mutant. ACE2 proteins of human/rhesus monkey and rat/mouse exhibited the highest and lowest receptor activities, respectively, with the other 10 ACE2s exhibiting intermediate activity. The implications of our findings are discussed in terms of the natural reservoir, zoonotic transmission, human-to-animal transmission, animal health, and animal model.

## RESULTS

### Human ACE2 serves as a functional receptor for SARS-CoV-2.

**FIG 2:**
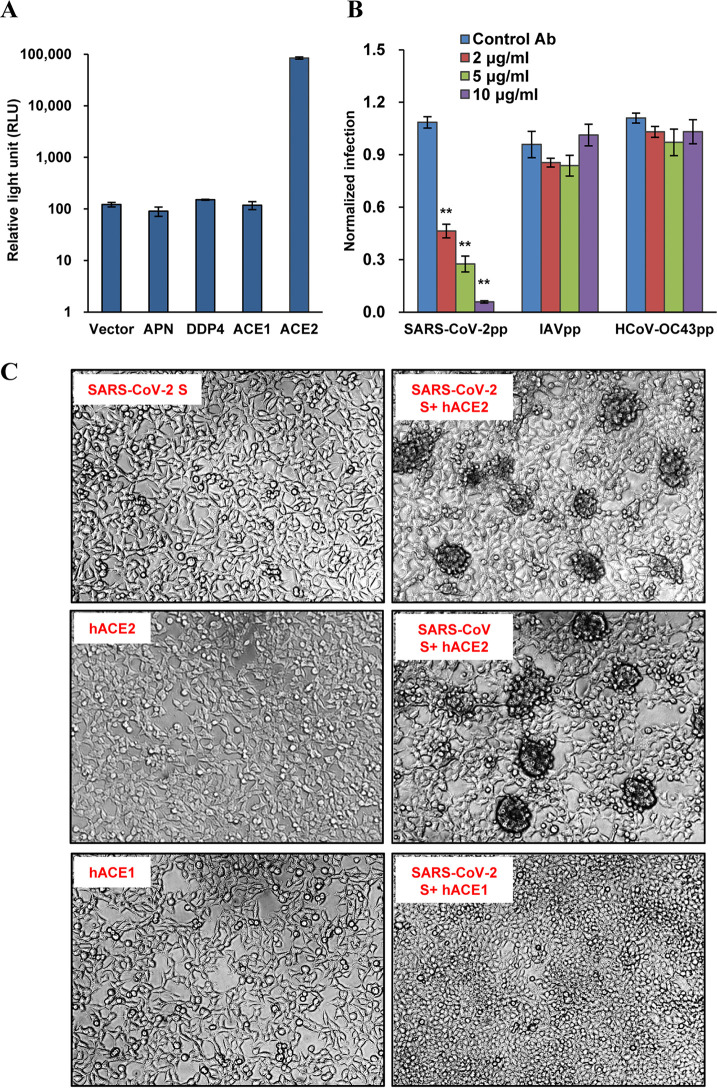
Human ACE2 served as a receptor for SARS-CoV-2. (A) ACE2 supported HIV-Luc-based pseudotyped virus entry. 293T cells were transfected with empty vector pcDNA3.1, APN (receptor for HCoV-229E), DDP4 (receptor for MERS-CoV), ACE1, or ACE2. At 48 h posttransfection, the cells were infected by SARS-CoV-2 S protein pseudotyped virus (SARS-CoV-2pp). At 48 h postinfection, luciferase activity was measured. Ab, antibody. (B) Human ACE2 antibody inhibited virus entry in a dose-dependent manner. 293T cells were transfected with ACE2. At 48 h posttransfection, the cells were preincubated with the indicated concentration of hACE2 antibody or control antibody (anti-IDE) for 1 h and then infected by pseudotyped virus particles of SARS-CoV-2, influenza virus A (IAVpp) or human coronavirus (HCoV) OC43 (HCoV-OC43pp) in the presence of the indicated concentration of hACE2 antibody or control antibody (anti-IDE) for another 3 h, and then the virus and antibodies were removed. At 48 h postinfection, luciferase activity was measured and normalized to the level of the control antibody for SARS-CoV-2pp. Error bars represent the standard deviations of the means from four biological repeats. (C) Syncytium formation assay. 293T cells transfected with a plasmid expressing the S protein of SARS-CoV-2 or SARS-CoV were mixed at a 1:1 ratio with cells transfected with a plasmid expressing ACE1 or ACE2. Twenty-four hours later, syncytium formation was recorded.

To examine the receptor activity of human ACE2 (hACE2) for SARS-CoV-2, we first established an HIV-based pseudotyped virus entry system. This system has been widely used in studies of coronavirus entry. To improve the expression level of S protein and the yield of pseudotyped virus, a codon-optimized S gene based on the sequence of isolate Wuhan-Hu-1 (2) was synthesized and used for production of pseudotyped virus as previously described for other human coronaviruses (HCoVs), including SARS-CoV, MERS-CoV, NL63, 229E, and OC43 (31, 32). The pseudotyped virus was then used to infect 293T cells transfected with either empty vector or a plasmid expressing APN (receptor for HCoV-229E), DDP4, (receptor for MERS-CoV), ACE1, or hACE2. At 2 days postinfection, the luciferase (Luc) activity was measured. As shown in Fig. 2A, only hACE2 was able to efficiently support virus entry. The entry of SARS-CoV-2, but not influenza virus A (IVA) or HCoV-43, was blocked by antibody against hACE2 in a dose-dependent manner (Fig. 2B). We also performed a syncytium formation assay to assess the membrane fusion triggered by hACE2-S binding. As shown in Fig. 1C, syncytium formation was seen only for cells expressing hACE2, but not hACE1, mixed with cells expressing the S protein of SARS-CoV-2 or SARS-CoV. These results confirm that hACE2 is the *bone fide* entry receptor for SARS-CoV-2.

### Multiple animal ACE2 orthologs serve as receptors for SARS-CoV-2 and a SARS-CoV-2 mutant with an S protein lacking the furin cleavage site.

**FIG 3:**
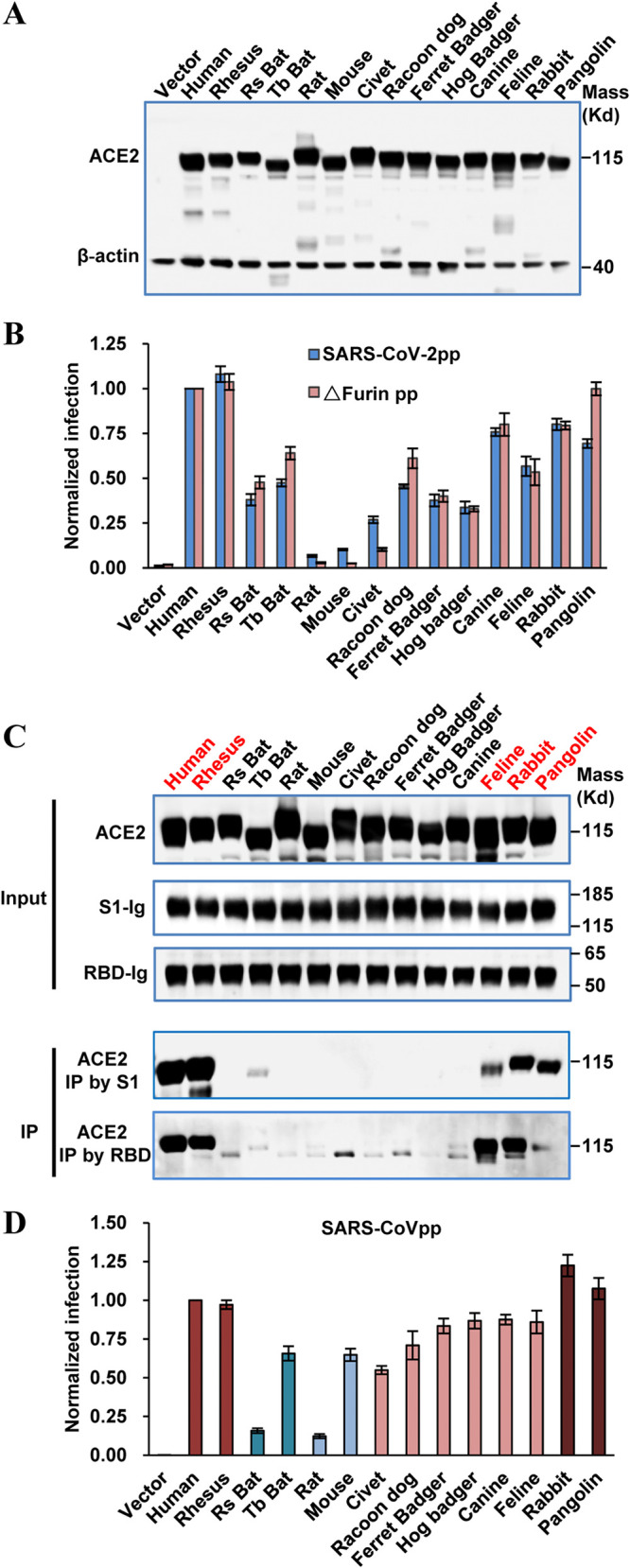
Multiple ACE2 orthologs served as receptors for SARS-CoV-2. (A) Transient expression of ACE2 orthologs in 293T cells. The cell lysates were detected by Western blot assay using an anti-C9 monoclonal antibody. (B) HIV-Luc-based pseudotyped virus entry. 293T cells were transfected with ACE2 orthologs. At 48 h posttransfection, the cells were infected by pseudotyped virus of wild-type SARS-CoV-2 or a mutant lacking furin (ΔFurin). At 48 h postinfection, luciferase activity was measured and normalized to that of human ACE2. Error bars represent the standard deviations of the means from four biological repeats. (C) IP assay. The upper panel shows the input of ACE2 protein with a C9 tag and S1 and RBD with an IgG Fc tag (S1-Ig or RBD-Ig). The lower panel shows the ACE2 pulled down by an S1-Ig or RBD-Ig fusion protein. (D) SARS-CoV spike-mediated entry. 293T cells were transfected with ACE2 orthologs. At 48 h posttransfection, the cells were infected by the pseudotyped virus of SARS-CoV. At 48 h postinfection, luciferase activity was measured and normalized to that of human ACE2. Error bars represent the standard deviations of the means from four biological repeats.

To test if other animal ACE2 orthologs can also be used as receptors for SARS-CoV-2, we cloned or synthesized ACE2 from rhesus monkey, Chinese horseshoe bat (R. sinicus), Mexican free-tailed bat (T. brasiliensis), rat, mouse, palm civet, raccoon dog, ferret badger, hog badger, canine, feline, rabbit, and pangolin. These animals were chosen as being either the proposed natural hosts for SARS-CoV-2 (bat and pangolin) (3, 10), intermediate hosts for SARS-CoV (civet and raccoon) (12), common animal models (rat, mouse, and monkey), or household pets (canine, feline, and rabbit). These ACE2 molecules were transiently expressed in 293T cells (Fig. 3A), which were then infected with pseudotyped virus particles of SARS-CoV-2 (SARS-CoV-2pp). The luciferase activity was measured and normalized to that of hACE2 (Fig. 3B). The results showed (i) that ACE2s of human and rhesus monkey were the most efficient receptors, (ii) that ACE2s of rat and mouse barely supported virus entry (<10% of hACE2), and (iii) that the levels of receptor activities of the other 10 animal ACE2s were between those of human/monkey and rat/mouse. Among these, ACE2s of canine, feline, rabbit, and pangolin could support virus entry at levels of >50% of the hACE2 level.

To examine receptor binding ability, we performed immunoprecipitation (IP) analysis by using both S1 and the receptor binding domain (RBD) as probes. Among the 14 different ACE2s tested, ACE2s from human, monkey, feline, rabbit, and pangolin exhibited significant and consistent association with S1 and RBD (Fig. 3C). Importantly, these ACE2s correspond to the group of ACE2s that supported the most efficient virus entry (Fig. 3B). The lack of significant entry reduction in 293T cells of furin mutant virus was likely due to the redundancy of cellular proteases, e.g., endosomal cathepsin, that promote membrane fusion in endosome. It has been proposed that a MERS-CoV mutant having an uncleaved S protein enters cells via the late endosome/lysosome (24). Two recent studies confirmed that furin cleavage of SARS-CoV-2 S protein was required for efficient entry into human lung cells (18, 33).

A striking difference between SARS-CoV-2 and animal SL-CoVs is the presence of a polybasic furin cleavage site at the S1/S2 boundary of the S protein (Fig. 1). Here, we generated a SARS-CoV-2 S gene mutant with the furin cleavage site deleted to mimic the bat SL-CoV CZ45. This S mutant has been previously demonstrated to express a full-length noncleaved S protein during biogenesis in cells (17). Pseudotyped virus with this mutant S protein was produced and used to infect ACE2-transfected 293T cells. Similar or slightly higher efficiencies were observed for the mutant S protein-mediated pseudoviral infections in cells transfected with all of the animal ACE2s, except for those of mouse, rat, and civet wherein the mutant S protein mediated a slightly lower efficiency of infection. Interestingly, pangolin ACE2 was now as efficient as hACE2 for supporting mutant virus entry (Fig. 3B).

We also tested the receptor usage of these 14 ACE2s by SARS-CoV (Fig. 3D). The results indicated that ACE2s of R. sinicus bat and rat were the poorest receptors (<20% of the hACE2 level), while the other ACE2s could support SARS-CoV entry at levels of >50% of the hACE2 level. Interestingly, ACE2s of rabbit and pangolin were even more efficient than hACE2 for supporting SARS-CoV entry. Together, these results demonstrated that SARS-CoV-2 and its mutant virus lacking furin cleavage site, as well as SARS-CoV, could use multiple animal ACE2s as receptors.

### Molecular basis of different ACE2 receptor activities.

**FIG 4:**
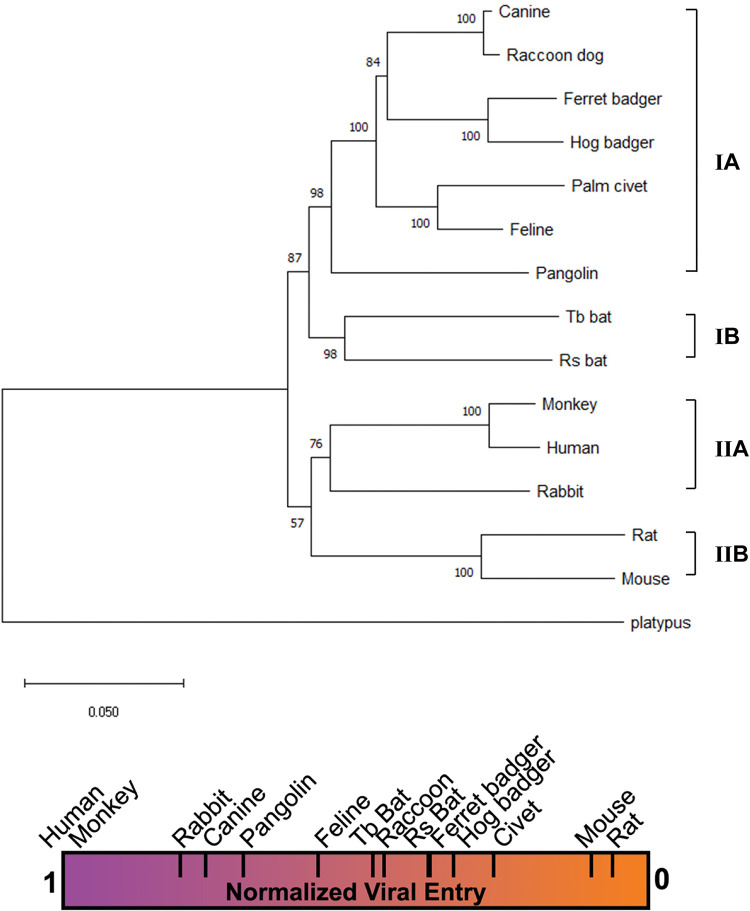
Phylogenetic clustering of ACE2s correlates with their receptor activities. At top is a phylogenetic tree of 14 ACE2s. The tree was constructed based on nucleotide sequences using the neighbor-joining method implemented in the program MEGA X. The percentages of replicate trees in which the associated taxa clustered together in the bootstrap test (1,000 replicates) are shown next to the branches. The tree was rooted by the ACE2 of platypus (Ornithorhynchus anatinus). The taxonomic orders into which these animals are classified are shown on the right-hand side of the tree. A heat bar summarizing the relative levels of pseudotyped virus entry supported by different animal ACE2s is shown below the tree.

To help understand the molecular basis of different ACE2 receptor activities, we first examined the overall sequence variation between these ACE2s. For this purpose, we constructed a phylogenetic tree based on the nucleotide sequences of ACE2s (Fig. 4). Interestingly, the phylogenetic clustering of ACE2s is correlated with their abilities to support SARS-CoV-2 entry. For example, ACE2s in subclade IIA (human, rhesus monkey, and rabbit) and IIB (rat and mouse) were the most efficient and poorest receptors, respectively, while ACE2s in clade I (from the remaining animals) were intermediate between subclades IIA and IIB. This correlation suggests that sequence variations that define species are responsible for observed differences in receptor activity.

**FIG 5:**
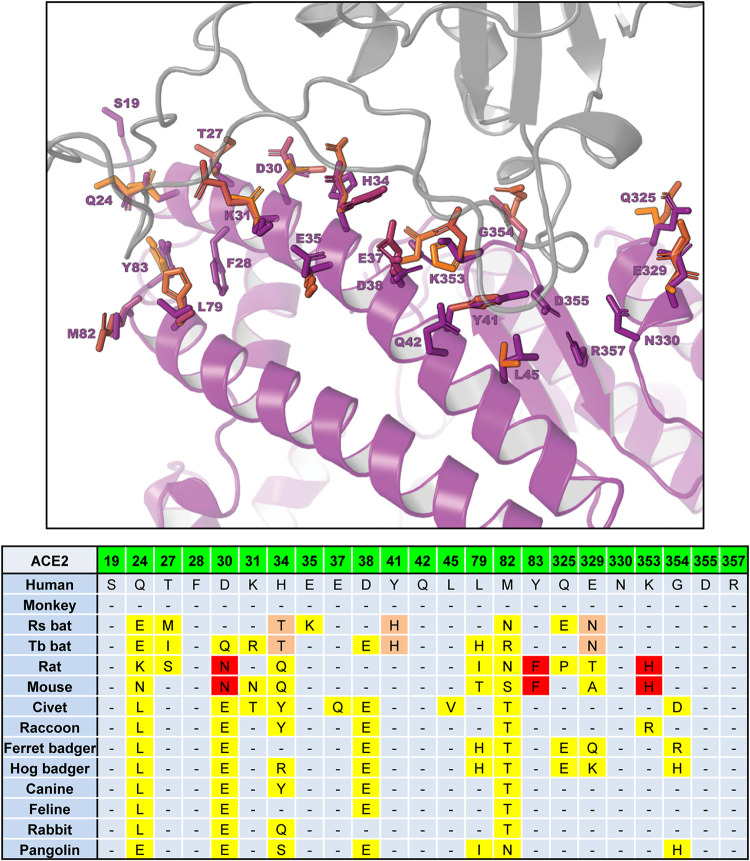
Critical RBD-binding residues in ACE2 orthologs. The top panel shows the 23 RBD-binding residues at the contact interface between hACE2 and the RBD of SARS-CoV-2. Human ACE2 (PDB accession no. 6VW1) in the bound conformation was extracted from the SARS-CoV-2 RBD/ACE2 complex and used as a template for homology modeling (16). Critical RBD-binding residues in ACE2 orthologs are shown in the bottom panel. Residue substitutions highlighted in red and orange are those unique to both mouse and rat ACE2s and to both bat species, respectively. Other residue substitutions are highlighted in yellow. Rs bat, Rhinolophus sinicus; Tb bat, Tadarida brasiliensis.

Next, based on the published crystal structures of the hACE2-RBD complex, we compared amino acid sequences of ACE2 receptors, focusing on 23 critical residues in close contact with the RBD of SARS-CoV-2 (16, 34, 35) (Fig. 5). Two obvious patterns were observed. First, hACE2 and rhesus monkey ACE2 are identical at all critical residues for RBD interaction. This explains why rhesus monkey ACE2 supported virus entry as efficiently as hACE2 (Fig. 3B). Second, since rat and mouse ACE2s support virus entry much less efficiently than other ACE2s, the three substitutions (D30N, Y83F, and K353H) that are seen only in rat and mouse ACE2s may be the key.

**FIG 6:**
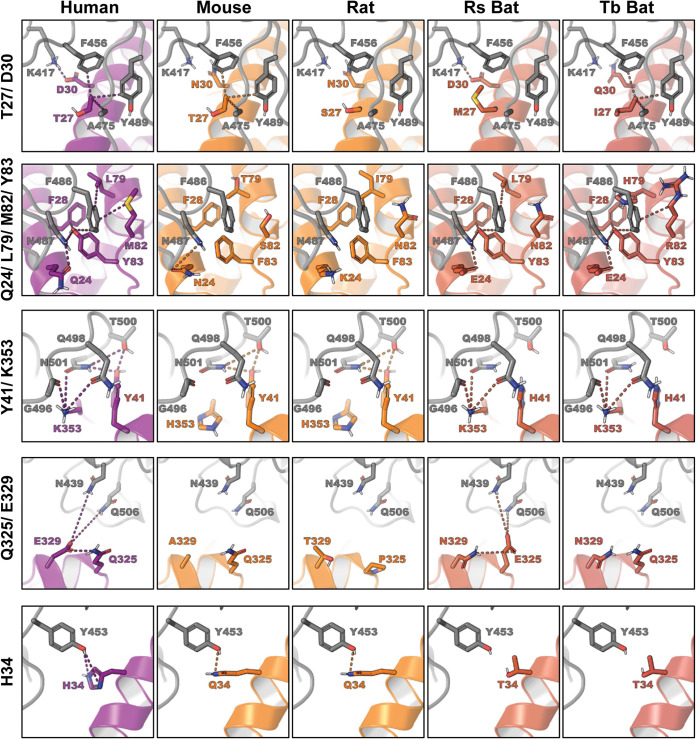
Structural models of key residue substitutions in ACE2 of mouse, rat, and bats. Human ACE2 (PDB accession no. 6VW1) in the bound conformation was extracted from the SARS-CoV-2 RBD/ACE2 complex and used as a template for homology modeling (16). ACE2 homology models were generated using the one-to-one threading algorithm of Phyre2 (63). The models were then aligned and compared to that of the intact SARS-CoV-2 RBD/ACE2 complex in PyMOL. Rs bat, Rhinolophus sinicus; Tb bat, Tadarida brasiliensis.

To further explain the different receptor activities, we used homology-based structure modeling to analyze the effect of residue substitutions at the atomic level. Structure models of 14 ACE2s were generated based on the crystal structure of SARS-CoV-2 RBD/ACE2 complex (16). The effects of critical residue substitutions were analyzed and are summarized in Table 1. Overall, the predicted effects of residue substitutions in ACE2s were consistent with corresponding receptor activities. ACE2s of rodents and bats are presented as examples of this analysis (Fig. 6).

**TABLE 1:**
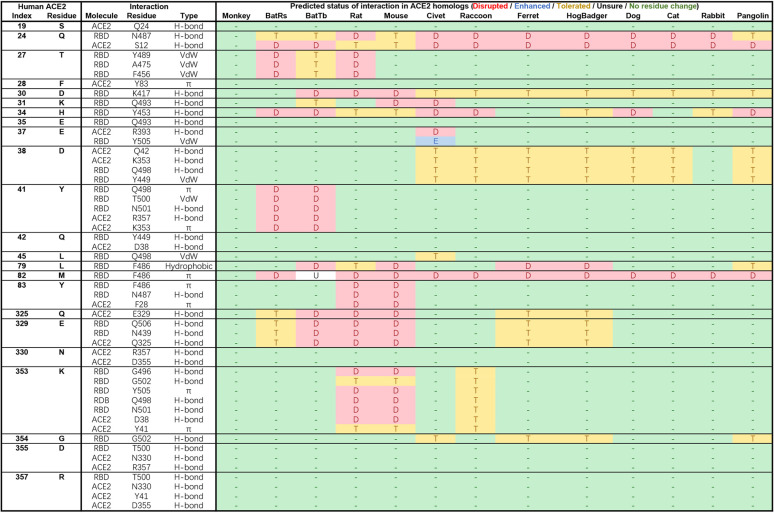
Predicted effect of clinical residue substitutions in ACE2 orthologs on the interaction with the RBD of SARS-CoV-2^*a*^

a H-bond, hydrogen bond; Vdw, Van der Waals force; π, π interaction; D, disruptive; T, tolerated; U, unsure; E, enhanced. The effects of residue substitutions were predicted by homology-based modeling analyses based on the crystal structure of the SARS-CoV-2 RBD/hACE2 complex (16).

First, we examined the rodent-unique substitutions D30N, Y83F, and K353H as they may play a key role in rat and mouse ACE2 inactivity. In humans, the residues at all three of these positions directly contact the RBD via hydrogen bonds. D30 contacts K417, Y83 contacts N487, and K353 appears to be at the center of a hydrogen bond network spanning seven RBD residues (Y449, G496, Q498, T500, N501, G502, and Y505) and eight ACE2 residues (D38, Y41, Q42, N330, K353, G354, D355, and R357). The D30N, Y83F, and K353H substitutions are all predicted to disrupt these interactions in rat and mouse ACE2 (Fig. 6). This is consistent with previous reports which pinpoint K353 as an important hot spot for binding of both SARS-CoV-2 (16) and SARS-CoV (36). It has been experimentally demonstrated that introduction of K353H into hACE2 significantly reduces binding to SARS-CoV S1; in contrast, introduction of H353K into rat ACE2 significantly increases binding to SARS-CoV S1 (37). Our homology models indicate that other residue substitutions may also be contributing to the low viral entry activity in mouse and rat ACE2s. Substitutions Q24N, Q27S, M82N, Q325P, and E329T in rat ACE2 and substitutions L79T, M82S, and E329A in mouse ACE2 are all predicted to disrupt interactions with RBD residues (Fig. 6 and Table 1).

Both bat ACE2s are also inefficient receptors for viral entry (Fig. 3B). Since the profile of residues at the receptor/RBD interface in bat is significantly different from residues of rat and mouse ACE2s, we examined other bat-specific residue substitutions that may be contributing to receptor dysfunction. There are 8 and 10 critical residue substitutions in the R. sinicus and T. brasiliensis bat ACE2s, respectively (Fig. 5). Among these, we examined the substitutions at positions Y41, H34, and E329 as they are seen only in bat ACE2s. The Y41H substitution in both bat ACE2s appears to be disrupting the same H-bond network that was disrupted by K353H in rat and mouse ACE2s. Although Y41 is not as centrally located in the H-bond network as K353, it directly contacts N501 from the RBD, which is the same residue that is stabilized by K353. A second interaction which appears to be disrupted in only bat ACE2s occurs at position H34. In humans, H34 forms an H-bond with Y453 from the RBD, which is broken through an H34T substitution in bat ACE2s. Finally, the bat-unique substitution E329N appears to be disrupting H-bonds connecting two ACE2 residues (E329 and Q325) and two RBD residues (N439 and Q506). In T. brasiliensis ACE2, all connections in the H-bond network are disrupted by the single E329N substitution; however, the H-bond network is predicted to be restored by an additional substitution, Q325E in R. sinicus. In addition, other residue substitutions, i.e., T27M and M82N in R. sinicus and D30Q and L79H in T. brasiliensis, are also disruptive (Fig. 6).

These results reveal that the poor and low receptor activities of rodent ACEs and bat ACE2s resulted from an interaction network broken by a key residue substitution, i.e., K353H in rodents and Y41H in bats, and additive disruptive effects by multiple residue substitutions.

## DISCUSSION

In this study, we examined the receptor activities of 14 ACE2 orthologs. The results suggested that wild-type (wt) and mutant SARS-CoV-2s lacking the furin cleavage site in the S protein could use ACE2s from a broad range of animal species to enter host cells. Below, we discuss the implication of our findings in terms of natural reservoir, zoonotic transmission, human-to-animal transmission, animal health, and animal model.

### Implications for natural reservoirs and zoonotic transmission.

Among the 14 ACE2s tested here, hACE2 and rhesus monkey ACE2 are the most efficient receptors, suggesting that SARS-CoV-2 has already been well adapted to humans. In addition, ACE2s of other animals, except mouse and rat, could also support SARS-CoV-2 entry (Fig. 3B). Although these data were obtained by using HIV-1-based pseudotyped virus, for ACE2 of R. sinicus bat, civet, and mouse, the data are consistent with *in vitro* infection data using infectious virus (3). Receptor usage by coronaviruses has been well known to be a major determinant of host range, tissue tropism, and pathogenesis (14, 38, 39). It is therefore reasonable to assume that SARS-CoV-2 would be able to infect all of these animals. As a matter of fact, several *in vivo* infection and seroconversion studies have confirmed that SARS-CoV-2 can infect rhesus monkey (40), feline, ferret, and canine (41, 42). Our findings are also in line with the concordance between ACE2 receptor usage by SARS-CoV pseudotyped virus and susceptibility of the animals to SARS-CoV infection. As shown in Fig. 3D, ACE2s of rhesus monkey, mouse, civet, ferret badger, raccoon, and feline could support SARS-CoV pseudotyped virus entry; concordantly, all of these animals are susceptible to native SARS-CoV virus infection (12, 43–46).

Among all of the wild animals that are potentially infected by SARS-CoV-2, bat and pangolin have already been proposed to be the natural reservoirs as closely related SL-CoVs have been identified in bats (2, 3, 5) and pangolins (8–11). A recent study has shown that bat SL-CoV RaTG13 could use hACE2 as a receptor, consistent with the presence of several favorable hACE2-binding residues (amino acids [aa] 455 and 482 to 486) in the receptor binding motif (RBM) of the S protein (Fig. 1) (16). For pangolin SL-CoVs, lineage PCoV-GD has only one noncritical amino acid substitution (Q483H) in the RBM compared to the sequence of SARS-CoV-2 (Fig. 1) (10). Therefore, PCoV-GD most likely can also use hACE2 and other animal ACE2s as functional receptors.

We also tested the receptor usage by a SARS-CoV-2 mutant that lacks the furin cleavage site at the S1/S2 boundary. Our result showed that the mutant virus behaved similarly to the wt virus. Namely, the entry of mutant virus could also be supported by the animal ACE2s that supported the entry of wt virus. This result is similar to that reported by another study that used the S gene mutant but in a murine leukemia virus (MLV)-based pseudotyped virus system (17) and that examined the role of furin cleavage during coronavirus infection. Furin cleavage is not essential for coronavirus-cell membrane fusion but enhances cell-to-cell fusion (19–22, 47). This could provide a certain level of advantage during infection. For example, in the quasispecies population of bovine CoV, a minor sequence variant with a polybasic furin-like cleavage site in the S2 subunit quickly dominated the population even after a single passage in cells (25). However, by using a pseudotyped virus system, which is a single-cycle infection system, we may not see the advantage. Still, our results unequivocally showed that SARS-CoV-2 without this cleavage site could use multiple animal ACE2s as receptors to enter cells. As there is a need to continuously search for potential intermediate hosts for SARS-CoV, results presented here can help significantly narrow down the scope of potential targets.

Collectively, our results highlight the potential of these wild animals to serve as natural reservoirs or intermediate hosts for SARS-CoV-2 and its progenitor, the risk of zoonotic transmission of animal SL-CoVs to human, and the necessity of virus surveillance in wild animals.

### Implications for human-to-animal transmission and animal health.

Among the animal species tested here, canine and feline are of special concern as they are often raised as companion pets. Our data indicate that ACE2s of canine and feline could support SARS-CoV-2 pseudotyped virus entry quite efficiently (>50% of the hACE2 level) (Fig. 3B), raising the alarming possibility of virus transmission from infected human to these pets or potentially vice versa. As a matter of fact, there was a recent report that a Pomeranian dog in Hong Kong tested weakly positive for SARS-CoV-2 while maintaining an asymptomatic state. The genome of the virus isolated from this dog has only three nucleotide changes compared to that of the virus isolated from two infected persons living in the same household, suggesting that this dog probably acquired the virus from the infected owners (48). Our results are further supported by two additional studies. One study showed that both dog and cat were susceptible to SARS-CoV-2 infection. While the virus replicated poorly in dogs, it replicated efficiently in cats and was able to transmit to unaffected cats that were housed with the infected animals (41). The other study revealed that 14.7% of cat serum samples collected in the city of Wuhan after the outbreak were positive for antibody against SARS-CoV-2, demonstrating that many cats were infected during the outbreak, most likely from infected humans in close contact (42). Domestic cats are also susceptible to SARS-CoV infection (43), and human-to-cat transmission was evident during the SARS-CoV outbreak in 2003 in Hong Kong (49). These findings were also in agreement with our results that ACE2s of cat and dog could serve as receptors for SARS-CoV (Fig. 3D).

As described above, it seems that dogs are not as susceptible as cats to SARS-CoV-2 (41, 48). Interestingly, this is in agreement with results from IP analysis that showed cat ACE2 could bind to S1 or RBD more efficiently than dog ACE2 (Fig. 3C). Structural models further suggest that, at the critical RBD-binding residues, dog and cat ACE2s share four substitutions (Q24L, D30E, D38E, and M82T), while dog ACE2 has an additional substitution, H34Y (Fig. 5). Based on structural modeling, both Q24L and M82T are predicted to be disruptive, while both D30E and D38E are tolerated (Table 1). H34Y in dog ACE2 is predicted to disrupt the hydrogen bond with Y453 of RBD (Table 1). These atomic interactions explain why dog ACE2 binds to S1 or RBD less efficiently than cat ACE2, and both are less efficient than human ACE2.

In addition to cat and dog, rabbits are also often raised as household pets. Our results indicate that rabbit ACE2 is an efficient receptor (Fig. 3B and C), suggesting that rabbit may be more susceptible to SARS-CoV-2 infection than cat.

Currently, there is no evidence that infected pets can transmit the virus back to humans; however, this may be possible and should be investigated. Out of an abundance of caution, it would be best when one is infected to have both human and pets quarantined and the pets tested as well.

### Implications for animal models.

Animal models are essential for the study of pathogenesis, vaccinology, and therapeutics of viral pathogens. Rodents are probably the most common and amenable animal models because of low cost, easy handling, defined genetics, and the possibility of scalability (50). However, our results showed that both mouse and rat ACE2s are poor receptors for SARS-CoV-2 (Fig. 3B and C), suggesting that they are probably resistant to infection. Actually, this has been verified by using infectious SARS-CoV-2 to infect mouse ACE2-transfected cells (3) or mice (51). Genetically engineered mice expressing hACE2 were previously developed as an animal model for SARS-CoV (52). This model has been tested recently for SARS-CoV-2 and found to be susceptible to SARS-CoV-2 infection and development of interstitial pneumonia (51), a common clinical feature of COVID-19 patients (53). Human ACE2-transgenic mice therefore represent useful animal models. However, because of the high demand for these mice and discontinuance of the model due to the disappearance of SARS-CoV in the human population after 2004, it is expected that this mouse model will be in short supply (54). Alternative methods should be sought to develop a mouse-adapted SARS-CoV-2 strain. Mouse-adapted SARS-CoV strains were developed by serial passage of virus in mice (55, 56). However, this method may not work for SARS-CoV-2 as mouse ACE2 still supports some entry for SARS-CoV (Fig. 3D) but not SARS-CoV-2. An alternative way to make a mouse-adapted SARS-CoV-2 strain could be achieved by rational design of the S gene. Based on the structural model, we know that receptor dysfunction of mouse ACE2 is due to disruptive D30N, L79T, M82S, Y83F, E329A, and K353H substitutions (Fig. 5 and 6 and Table 1). Therefore, by specifically introducing mutations into the RBM of the S gene, it may be possible to fully or partially restore interactions with these ACE2 substitutions. Consequently, the engineered virus may be able to efficiently infect wild-type mice.

To date, several animals (i.e., rhesus monkey, ferret, dog, cat, pig, chicken, and duck) have been examined as potential animal models for SARS-CoV-2 (40, 41). Although the rhesus monkey, ferret, and cat may seem to be promising candidates, none of them are perfect in terms of recapitulation of typical clinical features found in COVID-19 patients. Therefore, multiple animal models may be needed. Our results indicate that rabbit ACE2 is a more efficient receptor than other animal ACE2s for both SARS-CoV-2 and SARS-CoV (Fig. 3). Therefore, it may be worthy assessing rabbit as a useful animal model for further studies.

## MATERIALS AND METHODS

### Cell lines and antibodies.

293T cells and Lenti-X 293T cells were cultured in Dulbecco’s modified Eagle’s medium (DMEM; Gibco) (57). Growth medium was supplemented with 10% fetal bovine serum (FBS), 110 mg/liter sodium pyruvate, and 4.5 g/liter d-glucose. β-Actin antibody and C9 antibody were purchased from Sigma (A2228) and Santa Cruz (sc-57432), respectively. A polyclonal antibody against human ACE2 and anti-insulin-degrading enzyme (IDE) polyclonal antibody were purchased from R&D Systems (catalog numbers AF933 and AF2496, respectively).

### Construction of ACE2 plasmids.

ACE2s of human (Homo sapiens, GenBank accession number NM_001371415.1), civet (Paguma larvata, accession number AY881174.1), and rat (Rattus norvegicus, accession number NM_001012006.1) were cloned into a modified pcDNA3.1-cmyc/C9 vector (Invitrogen) as previously described (37, 58). ACE2 protein expressed from this vector has a c-*myc* tag at the N terminus and a C9 tag at the C terminus. An AgeI site was engineered downstream of the signal peptide sequence (nucleotides [nt] 1 to 54) of ACE2. ACE2s of Chinese ferret badger (Melogale moschata), raccoon dog (Nyctereutes procyonoides), Mexican free-tailed bat (Tadarida brasiliensis), rhesus monkey (Macaca mulatta), hog badger (Arctonyx collaris), New Zealand White rabbit (Oryctolagus cuniculus), domestic cat (Felis catus), and domestic dog (Canis lupus familiaris) were cloned into AgeI/KpnI-digested pcDNA3.1-cmyc-C9 vector as described previously (59). The nucleotide sequence of ACE2 of Chinese horseshoe bat (Rhinolophus sinicus, GenBank accession number KC881004.1) and pangolin (Manis javanica, GenBank accession number XM_017650263.1) were synthesized and cloned into the pcDNA3.1-cmyc/C9 vector.

### Construction of plasmids expressing S, S1, and RBD of SARS-CoV-2.

The nucleotide sequence of the SARS-CoV-2 S gene was retrieved from the NCBI database (isolate Wuhan-Hu-1, GenBank accession number MN908947). According to the method described by Babcock et al. (60), the codon-optimized S gene was synthesized and cloned into pCAGGS vector. The SARS-CoV-2 S gene mutant without the furin cleavage site at the S1/S2 boundary was generated by an overlapping PCR-based method as previously described (61). The S1 subunit (aa 14 to 685) and RBD (aa 331 to 524) were cloned into a soluble protein expression vector, pSecTag2/Hygro-Ig vector, which contains the human IgG Fc fragment and mouse IgG κ-chain leader sequence (61). The protein (S1-Ig or RBD-Ig) expressed is soluble and has a human IgG-Fc tag.

### Western blot assay.

As previously described, the expression levels of ACE2-C9, S1-Ig, and RBD-Ig fusion proteins were examined by Western blotting (61). Briefly, lysates or culture supernatants of 293T cells transfected with plasmid encoding ACE2 orthologs and S1-Ig or RBD-Ig were collected, boiled for 10 min, and then resolved by 4 to 12% SDS-PAGE. A polyvinylidene difluoride (PVDF) membrane containing the proteins transferred from an SDS-PAGE gel was blocked with blocking buffer (5% nonfat dry milk in Tris-buffered saline [TBS]) for 1 h at room temperature and probed with primary antibody overnight at 4°C. The blot was washed three times with washing buffer (0.05% Tween 20 in TBS), followed by incubation with secondary antibody for 1 h at room temperature. After three washes, the proteins bound to antibodies were imaged with a Li-Cor Odyssey system (Li-Cor Biotechnology).

### IP assay.

The association between Ig-fused S1 protein or RBD protein and ACE2 protein with a C9 tag was measured by immunoprecipitation (IP) according to a previously described method (61). Briefly, HEK293T cells were transfected with plasmid encoding ACE2 with Lipofectamine 2000 (Invitrogen). At 48 h posttransfection, the transfected 293T cells were harvested and lysed in phosphate-buffered saline (PBS) buffer containing 0.3% *n*-decyl-β-d-maltopyranoside (DDM; Anatrace). Cell lysates were incubated with protein A/G Plus-agarose (sc-2003; Santa Cruz) together with 4 μg of S1-Ig or RBD-Ig. Protein A/G-agarose-treated cells were washed three times in TBS–1% Triton X-100, resolved by SDS-PAGE, and detected by Western blotting using anti-C9 monoclonal antibody.

### Production of pseudotyped virus.

According to the standard protocol of calcium phosphate transfection, Lenti-X cells in 10-cm plates were cotransfected by 20 μg of HIV expressing luciferase (HIV-Luc) and 10 μg of CoV spike gene plasmid. At 48 h posttransfection, 15 ml of supernatant was collected and passed through a 0.45-μm-pore-size polyethersulfone (PES) filter. The purified virus was titrated with a Lenti-X p24 rapid titer assay (catalog no. 632200; TaKaRa Bio). The virus was stored at –80°C for future use.

### Virus entry assay.

Each well of 293T cells in a 96-well plate was transfected with 0.1 μg of ACE2 plasmid DNA according to the standard protocol for Lipofectamine 2000 (Invitrogen). At 48 h posttransfection, 150 μl of p24-normalized (10 ng) pseudotype virus was added into each well and incubated at 37°C for 3 h. The virus was then removed, and 250 μl of fresh medium was added to each well for further incubation. At 2 days postinfection, the medium was removed, and the cells were lysed with 30 μl/well of 1× cell lysis buffer (Promega) for 15 min, followed by addition of 50 μl/well of luciferase substrate (Promega). The firefly luciferase activities were measured by luminometry in a TopCount instrument (PerkinElmer). For each ACE2, four wells were tested in a single experiment, and at least three repeat experiments were carried out. The luciferase activity was expressed as the number of relative light units (RLU) and normalized to the level of human ACE2 for plotting.

### Syncytium formation assay.

293T cells with approximately 90% confluence on 12-well plates were transfected with 1.6 μg of plasmid DNA encoding the viral S gene or ACE2. At 24 h posttransfection, 293T cells expressing the S protein were mixed at a 1:1 ratio with 293T cells expressing ACE2 and plated on 12-well plates. Multinucleated syncytia were observed 24 h after the cells were mixed.

### Sequence analysis.

Multiple alignments of nucleotide or amino acid sequences of the spike gene of coronaviruses and ACE2 orthologs were performed using Clustal X (62). A phylogenetic tree was constructed based on the nucleotide sequences of animal ACE2s using the neighbor-joining algorithm implemented in MEGA X. The tree is drawn to scale with branch lengths in the same units as those of the evolutionary distances used to infer the phylogenetic tree. Evaluation of statistical confidence in nodes was based on 1,000 bootstrap replicates. Branches with <50% bootstrap values were collapsed. Platypus ACE2 (Ornithorhynchus anatinus, GenBank accession no. XM_001515547) was used as an outgroup.

### Homology-based structural modeling.

Human ACE2 (PDB accession no. 6VW1) in the bound conformation was extracted from the SARS-CoV-2 RBD/hACE2 complex and used as a template for homology modeling (16). ACE2 homology models were generated using the one-to-one threading algorithm of Phyre2 (63). The models were then aligned and compared to the intact SARS-CoV-2 RBD/ACE2 complex in PyMOL (PyMOL Molecular Graphics System, version 2.0, Schrödinger, LLC).

### Data availability.

Nucleotide sequences of the ACE2s of the domestic dog, Mexican free-tailed bat, Chinese ferret badger, raccoon dog, domestic cat, rhesus monkey, New Zealand White rabbit, and hog badger have been deposited in GenBank under accession numbers MT663955 to MT663962, respectively.
